# Limitations of Electromyography in the Assessment of Abdominal Wall Muscle Contractility Following Botulinum Toxin A Injection

**DOI:** 10.3389/fsurg.2019.00016

**Published:** 2019-04-09

**Authors:** Rodrigo Tomazini Martins, Kristen E. Elstner, Christian Skulina, Omar Rodriguez-Acevedo, John W. Read, Dominic B. Rowe, Nabeel Ibrahim

**Affiliations:** ^1^Hernia Institute Australia, Sydney, NSW, Australia; ^2^Department of Neurology, Macquarie University Hospital, Sydney, NSW, Australia; ^3^Department of Clinical Medicine, Faculty of Medicine and Health Sciences, Macquarie University Hospital, Macquarie University, Sydney, NSW, Australia; ^4^Department of Surgery, Macquarie University Hospital, Sydney, NSW, Australia

**Keywords:** abdominal wall, electromyography, complex ventral hernia, botulinum toxin A, lateral obliques, patient preparation, functional CT

## Abstract

**Purpose:** Pre-operative botulinum toxin A (BTA) injection of the lateral obliques aims to facilitate the closure of large ventral hernia defects and decrease the risk of repair breakdown during the critical healing phase. The exact duration of post-operative BTA effect and top-up timing in cases at high risk of recurrence remains uncertain. This study was designed to assess the value of electromyography (EMG) in determining the appropriate time for BTA top-up.

**Methods:** 56 patients underwent ventral hernia repair with pre-operative BTA infiltration of the lateral obliques. Eleven patients at high risk of recurrence considered suitable for BTA top-up were assessed post-operatively with both functional computed tomography (CT) and EMG. CT assessed segmental contractility of each muscle layer. Single-point EMG assessed the activity of individual muscle layers bilaterally in the anterior axillary line.

**Results:** CT showed (i) variable contractility of anterior and posterior muscle segments *prior to BTA injection*; (ii) absent or incomplete muscle paralysis in over half of all segments; (iii) *increased BTA effect* on progress scans; and (iv) non-uniform pattern of change in BTA effect between the anterior and posterior muscle. EMG demonstrated modest voluntary activity in most muscle layers. Compared to standard of reference (CT), EMG showed moderate sensitivity (0.62), poor specificity (0.48), poor accuracy (0.57), and incorrect grading in 71% of true positive results.

**Conclusions:** As BTA effect wanes, single-point EMG cannot reliably determine functional muscle status. A novel finding is that BTA-induced paralysis of the abdominal muscles may be remarkably non-uniform in degree, distribution and duration.

## Introduction

Botulinum toxin A (BTA) is an extremely potent neurotoxin synthesized by *Clostridium botulinum*. It blocks the neurotransmitter acetylcholine release at the neuromuscular junction of targeted muscles (1) and induces a flaccid paralysis that commences 2–3 days after injection (2), reaches maximum effectiveness after 2–4 weeks (3), and thereafter has a sustained effect that can last up to 6 months. The first therapeutic use of BTA was in neuro-ophthalmology to treat strabismus (4), but many other clinical indications have since been described (5), including abdominal wall reconstruction following “damage control” laparotomy (6, 7), primary closure of the acute open abdomen (8), and the management of post-operative pain following ventral hernia repair (9).

More recently, BTA injection has been used as a pre-operative “chemical component relaxation” technique to facilitate the repair of complex ventral hernias and reduce or eliminate the need for component separation (10). This procedure is performed under ultrasound guidance, and targets the External Oblique (EO), Internal Oblique (IO), and Transversus Abdominis (TA) muscles that comprise the lateral abdominal wall. Injections are performed at least 1–2 weeks prior to scheduled surgery to ensure maximum pre-operative effect (8, 11).

Profound relaxation of the injected abdominal wall segments causes an axial muscle elongation that facilitates successful midline closure and reduces repair line tension. The prolonged post-operative effect of BTA is also hypothesized to protect against subsequent incisional breakdown of the repair, and minimize risk of dehiscence during the early healing phase by limiting any physiological traction forces that would otherwise be exerted by the lateral abdominal muscles (10). In cases where the patient is considered to be at higher than average risk of hernia recurrence (i.e., with co-morbidities such as morbid obesity and diabetes, multiple failed previous repairs, collagen disorders or the use of immunosuppressive agents), a longer period of muscular paralysis by maintenance of “top-up” dose of BTA administered as the effect of the first dose begins to subside may provide additional protection. However, this management strategy would ideally require an objective method of determining the individual need for, and ideal timing of, any additional BTA dose top-up. Surface Electromyography (EMG) of the EO and IO muscles has previously demonstrated reduced maximal voluntary contraction on the side most affected by a hernia, compared to matched controls (12). Considering this, we investigated the potential use of invasive EMG to assess declining BTA effect on the 3 muscle layers, compared to the gold standard functional Computed Tomography (CT) in patients who underwent complex ventral hernia repair.

## Materials and Methods

A total of 56 complex multi-recurrent ventral hernia patients underwent pre-operative BTA injection. Informed consent was obtained in all cases. The EO, IO, and TA muscle layers on each side of the abdomen were then individually infiltrated under direct real-time ultrasound visualization as described in preliminary studies (9). Patients #1 to #32 received a total of 300 units of Botox® (or its equivalent of Dysport®) and patients #33 to #56 received a total of 200 units of Botox® (or its equivalent of Dysport®). In a sub-acute clinical outpatient setting using aseptic sterile technique, BTA was diluted with 0.9% sodium chloride (NaCl) to make 90 mL total and then divided equally into 6 syringes prior to infiltration at each abdominal wall location using 23G spinal needles. In all cases, injections of the individual muscles of the abdominal wall were performed as described by Farooque et al. (13). The reference puncture site was the anterior axillary line and needle was inserted into the posterior segment of each muscle. All patients underwent laparoscopic or laparoscopic-assisted mesh hernia repair by the same surgeon.

From the overall cohort, 11 patients (9 males and 2 females) were considered to be at a high risk of recurrence. Each of these underwent subsequent BTA maintenance dose injection from 3 to 6 months post-operatively. Immediately prior to “top-up” dose, the functional status of the abdominal wall was assessed with both functional CT and EMG, each performed on the same day. The working hypothesis was that any patient demonstrating some degree of voluntary muscle activity on EMG would benefit from a top-up injection.

**Figure 1:**
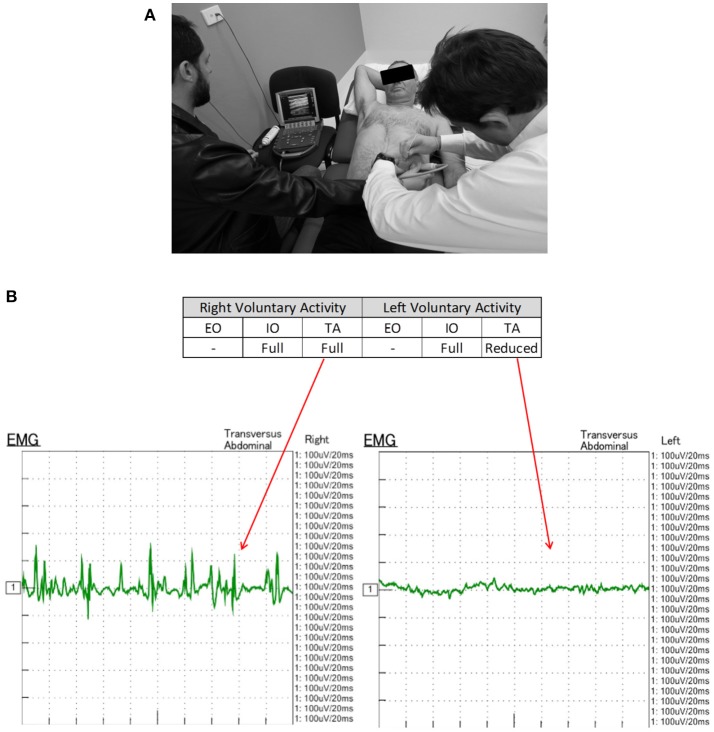
**(A)** EMG technique. With the patient in supine position, a monopolar needle is inserted into each of the targeted EO, IO, and TA muscles under direct ultrasound visualization prior to their individual functional assessment both at rest and during straining maneuvers. **(B)** EMG of patient #11 performed 6-10-16 shows electrical activity recorded from the right and left TA muscles during an attempted crunching maneuver. Muscle activity appears full on the right but reduced on the left. Corresponding 3 months post-operative crunching CT image (Figure 2) shows (i) a strongly contractile anterior segment but weak to non-contractile posterior segment of right TA muscle; and (ii) a moderately contractile anterior segment but only weakly contractile posterior segment of left TA muscle.

EMG was performed in supine position with all 3 muscle layers per side (EO, IO, and TA) individually tested at a single level in the anterior axillary line, yielding a total of 6 single-point muscle samples per case (one single puncture site on each side times 3 muscle layers—EO, IO, and TA, using a Nihon-Kohden EMG/EP device and either 50 or 75 mm long monopolar needles. Needle-tip placement was confirmed under real-time ultrasound guidance by an experienced sonographer using a portable Sonosite MTurbo scanner. Both spontaneous (resting) and voluntary muscle activity during an attempted “crunch” (sit-up) maneuver (12, 14) were recorded at each location by an experienced neurologist and EMG technician (Figure 1A). Muscle activity was graded qualitatively on the basis of size, shape, and frequency of the recorded electrical signals by an experienced neurophysiologist as either “Absent,” “Reduced,” “Moderate,” or “Full” activity.

**Figure 2:**
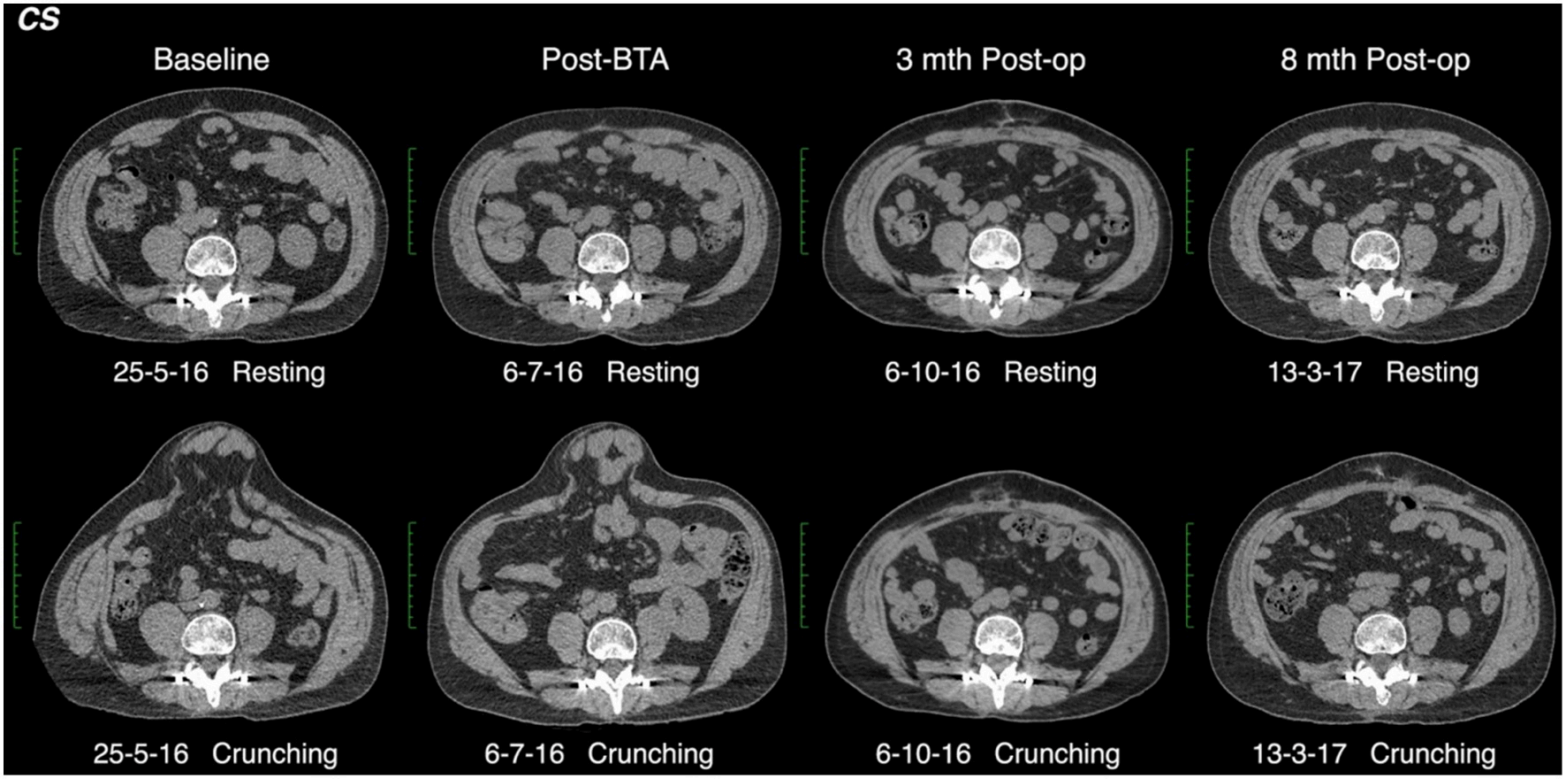
Functional CT methodology (patient #11). Comparison resting and crunching images obtained at the same axial level were used to measure the contractility index for each muscle segment.

All CT scans were “functional” examinations performed as helical image acquisitions with the abdominal wall both at rest and contracted during an attempted crunching maneuver (10, 13, 15). Radiation dose necessarily varied according to patient size, with the BMI in our series ranging from 22.6 to 54.0 kg/m^2^ (mean 32.6 kg/m^2^). For a non-contrast examination of abdominal wall comprised of a full length resting series and middle-third “crunching” series, the estimated *Dose Length Product* ranged from 319 to 2,670 mGy.cm (mean 1,172 mGy.cm). Muscle function on CT was graded quantitatively by an experienced radiologist by means of a “contractility index” (CI) defined as the percentage change in individual muscle layer thickness between resting and crunching scans measured at the same anatomical location. CI was measured separately for both the anterior and posterior segments of each muscle layer using the greatest muscle thickness that could be found in each segment. These measurements were also compared with both baseline pre-BTA pre-operative scans and early post-BTA pre-operative scans (Figure 2). The contractility of each muscle segment was graded as either Absent (CI = 0), Weak (CI ≤ 10%), Moderate (CI > 10% but ≤ 40%), or Strong (CI > 40%). Both CT and EMG necessarily *assumed* full patient compliance during the attempted crunching maneuvers used for each test.

## Results

The cohort was composed of 11 subjects (9 males) with a mean age of 62 ± 15 years and mean body mass index 29 ± 8 kg/m^2^. All BTA injections were carried out in a single session 1–4 weeks prior to hernia repair (mean 20 ± 18 days) and the abdominal wall assessment with both functional CT and EMG occurred at a mean 134 ± 25 days.

EMG performed with the abdominal muscles at rest detected no spontaneous motor activity at any of the locations tested in any patient. However, during attempted crunching maneuvers, all patients displayed some degree of voluntary muscle activity, particularly in the IO and TA layers (Table 1), with the degree of activity between sides being variable (Figure 1B).

**Table 1:** EMG results.

**Abdominal wall activity on EMG after operation**								
**Demographic data**			**Left voluntary activity**			**Right voluntary activity**		
**ID**	**Sex**	**Age (yr)**	**EO**	**IO**	**TA**	**EO**	**IO**	**TA**
1	F	64	-	-	Moderate	-	-	-
2	M	70	-	Moderate	-	Moderate	Moderate	Full
4	F	68	-	Reduced	Moderate	-	-	Reduced
5	M	53	-	Moderate	Moderate	-	-	Moderate
6	M	20	Moderate	-	-	-	-	Moderate
7	M	51	-	Reduced	Reduced	-	Reduced	Full
8	M	67	Reduced	Reduced	Reduced	-	Reduced	Reduced
9	M	63	-	Reduced	Reduced	-	Reduced	Reduced
10	M	62	Reduced	Moderate	Full	-	Reduced	Reduced
11	M	56	-	Full	Reduced	-	Full	Full

*Electrical activity within each muscle layer was graded qualitatively as either Absent (-), Reduced (+), Moderate (++), or Full (+++)*.

CT yielded a number of unexpected results. Firstly, baseline contractility of the EO, IO, and TA muscles in this group of patients with complex ventral hernias prior to any form of intervention was found to be variable and unpredictable, with up to 14% of either anterior or posterior muscle segments in any given layer being non-contractile *prior to BTA injection*. Secondly, when the anterior and posterior segments of all layers were assessed individually on early scans obtained *after BTA but prior to operation*, the muscle paralysis induced by BTA was absent in 30/132 (23%) segments, partial in 39/132 (29%) segments, and full in only 63/132 (48%) segments (Table 2). Thus, just over half of all segments in the muscles injected showed either absent or incomplete paralysis. Thirdly, comparison of the early post-BTA scans obtained prior to operation with subsequent progress scans done at the time of EMG showed an *apparent increase* in paralysis over time in just over one-third of all muscle segments. Lastly, the direction of change in BTA effect between the anterior and posterior segments of any given muscle layer was found to be concordant in only 28/66 (42%) and discordant in 26/66 (39%). For example, discordance was deemed present if the CI was found to diminish in the anterior segment while at the same time appearing to increase in the posterior segment *of the same layer* (or vice versa).

**Table 2:** Number of individual muscle segments showing full, partial or absent BTA effect on post-injection scans obtained *prior* to operation.

**BTA effect**	**200U dose**	**300U dose**
Full	29/72 (40%)	34/60 (57%)
Partial	27/72 (38%)	12/60 (20%)
None	16/72 (22%)	14/60 (23%)

Further data on BTA effect relative to dose administered is presented in Tables 2 and 3. Regardless of whether the anterior (right) or posterior (left) muscle segments of any given layer had been injected with BTA, there was no statistically significant difference in therapeutic effect on that layer for 300U (4.26 ± 2.51 vs. 4.18 ± 1.86 cm, *p* = 0.89) or 200U (3.84 ± 2.46 vs. 3.82 ± 2.64 cm, *p* = 0.98) dose of BTA, nor when assessing total muscle elongation (anterior vs. posterior muscle segments), regardless of the BTA dose (4.07 ± 2.48 vs. 4.02 ± 2.22 cm, *p* = 0.91).

**Table 3:** Comparison of the EMG and CT functional assessment of muscle layer contractility.

**Case**	**Muscle Layer**	**Right Side**			**Left Side**		
		**EMG activity**	**Segment tested**	**Concord EMG-CT**	**EMG activity**	**Segment tested**	**Concord EMG-CT**
1 (300U BTA)	EO	-	A	Y	-	A	Y
	IO	-	A	Y	-	A	Y
	TA	-	A	Y	++	A	N
2 (300U BTA)	EO	++	A	N	-	A	Y
	IO	++	A	N	++	A	N
	TA	+++	A	N	-	A	Y
3 (300U BTA) probable EMG side mislabeled	EO	+	A	Y	-	A	N
	IO	+	A	Y	-	A	N
	TA	++	A	Y	-	A	N
4 (300U BTA)	EO	-	A	N	-	A	N
	IO	-	A	N	+	A	Y
	TA	+	A	N	++	A	Y
5 (300U BTA)	EO	-	A	Y	-	A	N
	IO	-	A	Y	++	A	N
	TA	++	A	Y	++	A	N
6 (200U BTA)	EO	-	P	N	++	P	Y
	IO	-	P	N	-	P	N
	TA	++	P	Y	-	P	N
7 (200U BTA)	EO	-	A	N	-	A	N
	IO	+	A	Y	+	A	Y
	TA	+++	A	Y	+	A	Y
8 (200U BTA)	EO	-	A	Y	+	A	Y
	IO	+	A	Y	+	A	N
	TA	+	A	N	+	A	N
9 (200U BTA)	EO	-	A	N	-	A	N
	IO	+	A	Y	+	A	Y
	TA	+	A	Y	+	A	Y
10 (200U BTA)	EO	-	A	N	+	A	Y
	IO	+	A	Y	++	A	Y
	TA	+	A	N	+++	A	Y
11 (200U BTA)	EO	-	A	N	-	A	N
	IO	+++	A	Y	+++	A	Y
	TA	+++	A	Y	+	A	Y

*The grading of activity within each muscle layer on EMG (-, +, ++, or +++) is rated for concordance (Yes, Y or No, N)*.

The full correlative EMG-CT dataset is given in Table 3. The calculation of EMG performance relative to CT was underpinned by two basic *assumptions:* (i) that a percentage *change* in the contractility index on CT of <5% was within the range of human measurement error and therefore likely to reflect no change at all; and (ii) that the EMG grading of “Reduced,” “Moderate,” and “Full” muscle activity corresponded with the CT contractility index grading of “Weak,” “Moderate,” and “Strong,” respectively (Table 4). One patient (#3) was excluded from the final analysis due to a possible error in the labeling of sides (as it was noted that the CT findings in this case would exactly match those on EMG *provided* the labeling of sides on EMG was reversed). For the remaining 10 patients included in the final analysis, EMG assessment of individual muscle function was true positive for 24 layers, true negative for 10 layers, false positive for 11 layers, and false negative for 15 layers. Thus, EMG in this cohort had an overall sensitivity of 62%, specificity of 48%, positive predictive value of 69%, negative predictive value of 40%, and accuracy of 57%. EMG incorrectly graded muscle function in 26/60 (43%) sampled layers.

**Table 4:** CT contractility index (CI) of the same segment, presented on Table 4, on progress “post-BTA scan 2” (noting “post-BTA scan 1” as the earliest post-BTA measurements obtained pre-operatively).

**Case**	**Muscle layer**	**Right side CT contractility index**						**Left side CT contractility index**					
		**Anterior**			**Posterior**			**Anterior**			**Posterior**		
		**Pre BTA**	**Post BTA 1**	**Post BTA 2**	**Pre BTA**	**Post BTA 1**	**Post BTA 2**	**Pre BTA**	**Post BTA 1**	**Post BTA 2**	**Pre BTA**	**Post BTA 1**	**Post BTA 2**
1 (300U BTA)	EO	31	−42	−52	−14	−3	6	14	−24	−2	2	−22	92
	IO	15	−36	−54	−24	−11	−18	44	−4	−15	6	6	70
	TA	−1	−17	−28	12	34	−12	25	−5	−2	30	−24	−11
2 (300U BTA)	EO	26	−17	−11	45	−12	2	−4	−19	−23	16	7	−13
	IO	24	−29	−46	114	16	−9	5	−46	−22	43	12	4
	TA	−3	18	−2	−7	−29	−25	−14	−32	−13	−12	−25	10
3 (300U BTA) probable EMG side mislabeled	EO	−2	−22	7	−8	−14	158	2	−22	17	18	2	40
	IO	10	−15	2	4	9	−15	35	0	82	17	142	84
	TA	31	−38	30	36	−7	21	18	−18	63	37	55	31
4 (300U BTA)	EO	176	115	11	24	75	67	39	−10	20	38	40	19
	IO	41	30	7	68	108	99	95	−15	23	65	78	84
	TA	90	−4	80	87	33	−19	52	−2	5	174	49	16
5 (300U BTA)	EO	36	24	−35	29	22	35	23	−28	2	35	−29	2
	IO	57	−47	−63	64	78	90	97	−12	−48	27	56	107
	TA	10	−38	9	55	8	56	39	−31	−38	58	7	23
6 (200U BTA)	EO	25	−5	7	18	−16	15	40	−2	4	−1	−2	4
	IO	36	−25	25	28	46	37	30	17	18	69	22	27
	TA	67	42	48	48	14	24	145	−31	−5	71	66	4
7 (200U BTA)	EO	13	5	2	18	37	48	18	8	3	0	23	53
	IO	−2	−3	87	32	53	63	−28	−6	65	3	32	34
	TA	3	42	16	33	67	77	53	69	66	52	50	89
8 (200U BTA)	EO	25	−5	−9	37	97	14	23	12	1	24	12	31
	IO	75	14	22	−38	−16	−15	27	−21	−66	−12	−34	−4
	TA	79	A	N	6	−1	−3	36	5	−54	−10	−35	−61
9 (200U BTA)	EO	13	5	N	54	11	5	25	−14	1	91	0	7
	IO	88	24	107	18	−33	20	62	−39	37	58	−38	14
	TA	122	22	43	85	7	−16	34	−8	17	54	−11	−44
10 (200U BTA)	EO	28	7	8	26	24	17	11	−6	10	58	50	21
	IO	90	8	58	110	44	3	102	57	38	19	67	79
	TA	24	0	−5	160	−8	30	18	3	69	107	39	110
11 (200U BTA)	EO	24	−25	23	28	4	29	26	−29	14	56	74	14
	IO	119	−53	16	70	15	46	56	−40	55	82	72	86
	TA	28	−41	68	40	−24	−14	85	−6	15	115	−12	6

*Positive CI values indicate an increase in muscle thickness on crunching (i.e., declining BTA effect with now contractile muscle). Negative CI values indicate a decrease in muscle thickness on crunching (i.e., persisting BTA effect with flaccid non-contractile muscle)*.

## Discussion

Bueno-Lledo and colleagues reported the pre-operative injection of BTA for large incisional hernia repairs under EMG and ultrasound guidance (16) but did not describe any subsequent post-operative role for EMG. Other groups made use of EMG during BTA injection aiming to identify the location of maximal muscle activity (6, 17). Rodriguez-Acevedo et al. demonstrated that there was no statistically significant difference in muscle elongation gain per side between the therapeutic effect of a 300 units and 200 units dose of BTA (15). Our investigation was therefore performed to assess the potential value of post-operative EMG in gauging the reversal of muscle paralysis induced by pre-operative BTA injection in patients who have undergone complex ventral hernia repair and may benefit from an additional top-up injection. The theoretical advantages of this approach were an anticipated high sensitivity for the detection of recovering muscle activity and the avoidance of radiation exposure associated with CT (the alternative diagnostic strategy).

However, we unexpectedly found single-point EMG sampling to be an unreliable test for the return of muscle function when compared against the chosen reference standard of functional CT. A number of factors may have contributed to the poor overall sensitivity and accuracy of EMG. Chief amongst these was unanticipated variability in the overall degree, spatial distribution, peak timing and total duration of BTA effect. Similarly, the absence of EMG guided BTA injection may have failed to identify fibrotic or denervated areas, as a consequence of previous surgeries (16, 18). As no patient in our series showed complete paralysis of all segments of all muscles on CT after BTA injection, any finding of electrical activity on post-operative EMG could not be taken in isolation as a reliable indicator of waning BTA effect (EMG was falsely positive in 11 out of 30 tested layers). Conversely, the complete absence of electrical activity on post-operative EMG in any tested muscle could be false negative if the electrical sample site did not include the functioning segment of that same muscle (EMG was falsely negative in 15 out of 30 tested layers). Other technical factors that could adversely affect EMG performance included inaccurate placement of the EMG needle tip (e.g., patient #1 had abdominal wall scarring from multiple previous surgeries that obscured the ultrasound identification of individual muscle layers on one side), uncertainty concerning the exact segment that was sampled by EMG (e.g., unlike all other cases in our series, the clinically chosen sample point of anterior axillary line in patient #6 corresponded on CT with the boundary between the anterior and posterior halves of the lateral abdominal wall muscles, and it was estimated that an inferiorly directed needle tip would more likely have sampled the posterior than the anterior segments of these muscles), poor compliance, consistency or understanding of maneuver when the patient was asked to perform “crunch” maneuvers during testing, and the arguable validity of our arbitrarily devised grading schemes for muscle function on EMG and CT as being fair and comparable.

Other variables were also present in our small patient series. In addition to BTA injection, a minimally invasive form of External Oblique Release (EOR) was performed in three patients (#1, #3, and #10) at the time of hernia repair. This could have adversely influenced EMG performance if the needle tip was inserted into a zone of post-surgical fibrosis where no muscle activity would be recorded. One patient also underwent progressive pre-operative pneumoperitoneum (PPP) as an adjunctive procedure, although no adverse impact on EMG performance would be anticipated in this case. Additionally, Ozkan et al has already reported nerve dysfunction caused by 2 types of commonly used mesh 4 weeks post hernia surgery (19).

Limitations also apply to the assessment of muscle function by CT. Firstly, full patient compliance during attempted “crunch” maneuvers is an assumption potentially flawed by either patient misunderstanding or incapacity. Secondly, CT may underestimate the degree to which BTA effect has reversed due to increased lateral traction forces on the EO, IO, and TA muscles after surgical restoration of the abdominal midline (i.e., increased tension will tend to maintain a longer and thinner muscle despite any increase in resting muscle tone as BTA effect wanes). Indeed, this phenomenon may explain other seemingly paradoxical results in our study. The apparent increase in paralysis over time in just over one third of total injected muscle segments, as well as the discordant direction of change in BTA effect between anterior and posterior segments of any given muscle layer, might be explained as a consequence of increased lateral traction force exerted by an increasingly active muscle segment on its still paralyzed companion muscle segment. Lastly, if the boundaries used to measure muscle thickness are unclear and/or the thickness of any particular muscle is quite small, there is potential for measurement error on CT when calculating the contractility index.

Radiation dose is an important consideration when utilizing CT as a diagnostic test. The mean dose associated with our series was above the reference level (DLP 600 mGy.cm) for abdomen published as a guideline by the Australian regulatory authority (ARPANSA), but nevertheless unavoidable due to the large physical size of complex ventral hernia patients. We considered the clinical benefit of CT in these difficult cases to outweigh the small attendant radiation risk.

Our results also provide new insights on both abdominal muscle function in chronic ventral hernia patients and the therapeutic effect of BTA. Firstly, the functional contractility of segments within the EO, IO, and TA muscles in this group of patients with complex ventral hernias was found to be variable and unpredictable prior to any form of medical intervention (including up to 14% of either anterior or posterior muscle segments in any given layer being non-functional *before* BTA was injected). However, in the absence of any control group, it is not conclusive that ventral hernia defects are associated with segmental non-function. Secondly, we have shown that BTA injection of the lateral abdominal muscles has (i) a typically non-uniform and incomplete paralyzing effect at the doses given, (ii) no significant difference in the paralyzing effect on any given layer whether the anterior or posterior segments are injected, and (iii) no significant difference in therapeutic effect between 200 and 300 units doses. However, these non-uniform findings were similarly reported by Daurova et al in patients with ventral midline hernia, where 60% of them did not show full muscular recovery on EMG post hernia surgery (20).

Other weaknesses of our study include a small sample size and the lack of any pre-operative EMG obtained at baseline (required to show any electrical change in muscle activity over time).

## Conclusion

As BTA effect wanes after initial injection, the overall functional status of any given abdominal muscle layer cannot be reliably determined from single-point EMG sampling alone in this cohort. An important contributing factor is the novel and unexpected finding that BTA-induced paralysis of the abdominal muscles may be remarkably non-uniform in degree, distribution and duration. Further, studies with larger cohorts are required and may provide more consistent data.

## Ethics Statement

The study was exempt from Ethics approval according to Prof Anthony Eyers, Chairman of the Ethics Committee at Macquarie University Hospital, Sydney, Australia. However, all enrolled subjects gave verbal consent to the procedures involved in this project.

## Author Contributions

NI and JR conceived the study. RT, CS, and JR collected the data. RT, KE, and OR-A analyzed the data and drafted the manuscript. RT and JR prepared the tables and figures. All authors reviewed the manuscript and approved the final version.

### Conflict of Interest Statement

JR receives financial remuneration from Medicare for radiologist work. The remaining authors declare that the research was conducted in the absence of any commercial or financial relationships that could be construed as a potential conflict of interest.
